# Jaw biodynamic data for 24 patients with chronic unilateral temporomandibular disorder

**DOI:** 10.1038/sdata.2017.168

**Published:** 2017-11-07

**Authors:** José López-Cedrún, Urbano Santana-Mora, María Pombo, Amaya Pérez Del Palomar, Víctor Alonso De la Peña, María Jesús Mora, Urbano Santana

**Affiliations:** 1 Oral and Maxillofacial Surgery Service, University Hospital of La Coruña, 15006 La Coruña, Spain; 2 Department of Surgery and Medical-Surgical Specialties of the University of Santiago de Compostela, Santiago de Compostela, 15782 La Coruña, Spain; 3 Biomaterials Group, Aragon Institute of Engineering Research, University of Zaragoza, 50018 Zaragoza, Spain; 4 Oral and Maxillofacial Surgery Service, University Hospital of Santiago de Compostela, Santiago de Compostela, 15706 La Coruña, Spain

**Keywords:** Musculoskeletal abnormalities, Diagnosis, Musculoskeletal system

## Abstract

This study assessed 24 adult patients, suffering from severe chronic unilateral pain diagnosed as temporomandibular joint (TMJ) disorder (TMD). The full dentate patients had normal occlusion and had never received an occlusal therapy, i.e., were with natural dental evolution/maturation. The following functional and dynamic factors were assessed: (1) chewing function; (2) TMJ remodeling or the condylar path (CP); and (3) lateral jaw motion or lateral guidance (LG). CPs were assessed using conventional axiography, and LG was assessed by K7 jaw tracking. Seventeen (71%) of the 24 (100%) patients consistently showed a habitual chewing side. The mean (standard deviation [SD]) of the CP angles was 47.90 (9.24) degrees. The mean (SD) of the LG angles was 42.95 (11.78) degrees. Data collection emerged from the conception of a new TMD paradigm where the affected side could be the habitual chewing side, the side with flatter lateral jaw motion or the side with an increased CP angle. These data may lead to improved diagnosis, therapy plans and evolution in TMD patients.

## Background & Summary

The chief complaints of patients with chronic temporomandibular joint (TMJ) disorder (TMD) are preauricular pain that increases during the chewing of hard foods and limited jaw opening. Although both sides can be affected, symptoms mainly affect one side^1^. Symptom etiology is unknown^2,3^. One study found that the overall prevalence of severe TMD pain was 6.3% among women and 2.8% among men^4^. The diagnosis of TMD is most often based on patient history and a physical examination^5–8^.

**Figure 1:**
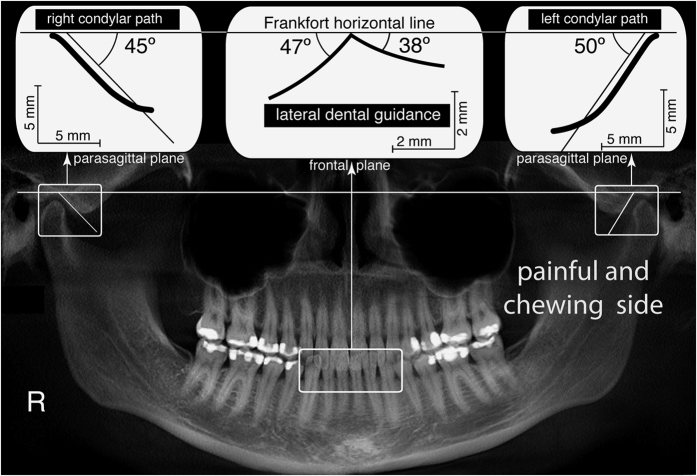
Panoramic X-ray of a patient suffering from chronic severe left pain diagnosed as temporomandibular joint disorder. This X-ray shows sided differences in TMJ eminence remodeling and includes 2D free diagrams of lateral dental guidance and condylar path angle recordings.

A preferred chewing side^9,10^ is a more common condition in subjects with TMD symptoms^1,11–14^ than in young nonpatients^3,15^. Experimental animal studies^10,16^ have shown that a habitual chewing side contributes to TMJ remodeling. Associations have been demonstrated among specific TMJ characteristics (e.g., steeper condylar path [CP] angles), dental occlusion, remodeling (e.g., flatter lateral guidance [LG] angles) (Fig. 1), and habitual chewing on the affected side in patients with chronic unilateral TMD symptoms^7^; however, no studies have assessed these characteristics in subjects with normal occlusion who have never undergone an occlusal therapy, such as orthodontics or prosthodontics. This aspect could be critical because occlusal therapies can suddenly change LG angles and plausibly alter chewing function.

Human jaw biodynamics during jaw closure, chewing or lateral motion (functional or parafunctional) are guided by a tripod consisting of the two TMJs and dental guidance. The path followed by the jaw during lateral motion or chewing can be assessed by measuring recordings of the condyles or dental guidance.

This cross-sectional double-blind study found potentially relevant TMD-related outcomes in a homogeneous group of patients diagnosed with TMD and suffering from considerable chronic (over 6 months) unilateral pain (4 ≤ pain intensity ≤ 9, according to a visual analog scale [VAS] scale on which 0 is no pain and 10 is the worst possible pain). The affected painful side, chewing function, and CP and LG angles were assessed in patients with normal dental articulation (occlusion) and substantial self-reported chronic unilateral TMD pain who had never undergone any kind of occlusal therapy, i.e., with natural dental evolution/maturation.

These data could facilitate the diagnosis of potential contributors to clinical or preclinical TMD conditions and/or altered chewing function in order to establish therapy plans and/or early/preventive strategies and to implement mathematical models of the stomatognathic system of TMD patients.

The primary biological characteristics of patients (Data Table 1, Data Citation 1), raw data from the CP (Data Table 2, Data Citation 1) and LG (Data Table 3, Data Citation 1) angles, and asymmetry indexes of the CP and LG angles (Data Table 4, Data Citation 1), and reliability of the measurements of the CP and LG angles (Data Table 5, Data Citation 1) can be found in Microsoft Excel.xlsx format.

## Methods

### Ethics statement

This study was approved by the Autonomic Committee of the Research Ethics of Galicia: CAEI approval number 2009/017; updated on November 29, 2013. Informed consent was obtained from all participants, and the data released here are consistent with the consent obtained.

### Subjects

This study assessed patients with chronic (over 6 months) unilateral pain diagnosed as TMD according to the Diagnostic Criteria/TMD^8^ who were referred to a public hospital for tertiary care. Their self-reported pain (4 ≤ intensity ≤ 9)^17^ was rated according to a VAS scale^18^ on which 0 is no pain and 10 is the worst possible pain. They had clinically normal dental articulation (main inclusion criterion). Moreover, participants met the inclusion criteria recommendations stated in the IMMPACT guidelines^17^. The exclusion criteria also followed the IMMPACT guidelines^17^. In addition, patients suffering from bilateral TMD pain or who were being treated with orthodontic and/or prosthetic therapy were excluded.

Twenty-four patients who fulfilled the abovementioned selection criteria were randomly selected.

Data included in the text of the rest of the Data Records section, not data in the data tables themselves, are presented as the mean (s.d.).

### Chewing function

Chewing side was assessed by observing gum chewing (first and seven subsequent strokes), performing kinesiography and conducting an interview. To assess the current and former chewing side, the patients answered the question, ‘Do you use one particular chewing side to eat?’ with ‘No, alternate sides,’ ‘Yes, the right side,’ ‘Yes, the left side,’ or ‘I don’t know.’ Consistent use of the same chewing side in all tests was considered to indicate a habitual chewing side, whereas the use of a different chewing side in any test was considered to represent alternate chewing, with the exception of the patient answering, ‘I don’t know’ in the interview.

### CP angles

**Figure 2:**
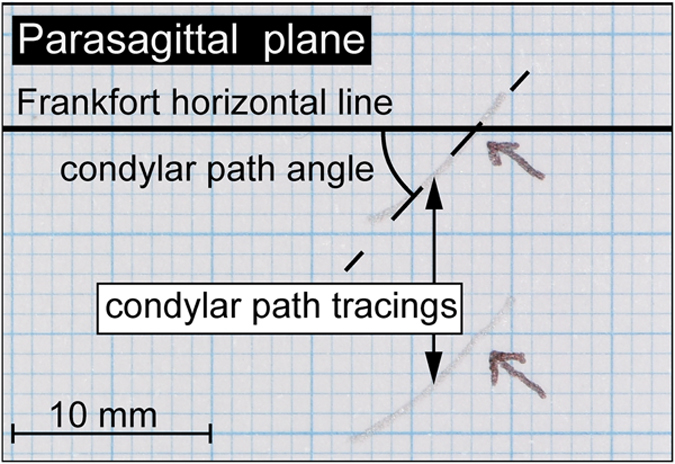
Original diagram showing how the left condylar path angle can be measured Angle value can be assessed using ImageJ software.

Parasagittal plane CP tracings were recorded using a kinematic Gerber’s face-bow (Kit Registier Ausrustung ‘C;’ Condylator service, Zurich, Switzerland)^19^. Four traces were recorded on each side. The recordings were telephotographed perpendicular to the card and analyzed using ImageJ software. Angles between the CP tracings and the Frankfort horizontal line (3 mm forward from the posterior passive condyle position) were measured by two experienced researchers. Although the start point can usually be recognized in a given tracing, ideally, the beginning of the recording should be marked during a clinical procedure (Fig. 2, arrows). Arrows indicate the start point when the mandible moves from the central position to the opposite side. The first part of the top of the tracings corresponds to ipsilateral jaw movement and should be not considered for measurements; the subsequent 3 mm after the arrow are considered.

### LG angles

**Figure 3:**
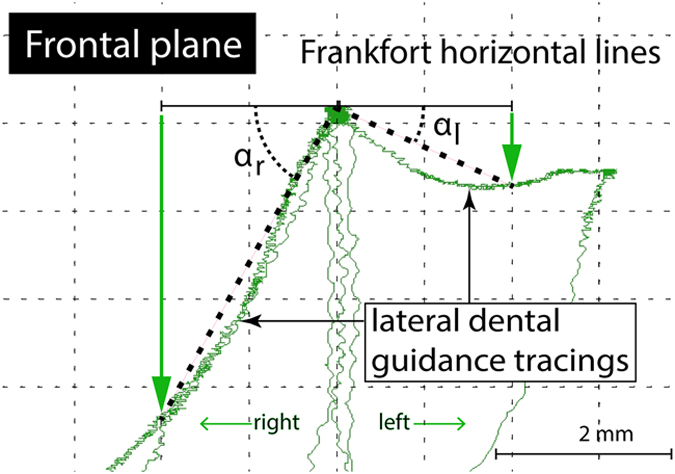
Frontal plane lateral guidance kinesiography from a temporomandibular joint disorder participant with chronic left pain. αr, right side lateral dental guidance; αl, left side lateral dental guidance.

Frontal plane LG recordings were conducted using a jaw-tracking device (K7 electrodiagnostic system; Myotronics Inc., Kent, WA, US), and α_r,l_ angles were measured from the maximal intercuspal position to the point of 2 mm of lateral motion^20,21^ (Fig. 3).

## Data Records

### Data storage

Raw data are displayed in the Dryad Digital Repository [Data Citation 1].

### Chewing side

Seventeen (71%) of the 24 (100%) patients with unilateral TMD pain showed one consistent habitual chewing side. This habitual chewing side tended to be the affected side (the Pearson chi-squared test: 10.578; *P* = 0.001; Fisher's exact test [two-tailed]: *P* = 0.002; Kappa value: 0.767, *P* = 0.001). The risk estimate was 4.5 (95% confidence interval [CI]: 1.326 to 15.277).

### CP or TMJ remodeling assessed by axiography recordings

The CP angles of two TMJs from two participants were excluded due to aberrant form/motion. Specifically, the magnetic resonance imaging (MRI) of a man’s left TMJ (symptomatic side) showed condylar flattening, sclerosis of the dome, an osteophyte, and anterior disc displacement without reduction. The other case of TMJ exclusion was a woman who presented with the absence of a disc in the right TMJ (symptomatic side). (Table 1).

The global mean was 47.90 (9.24) degrees, range 34 to 70. The Kolmogorov-Smirnov test did not reject the normal distribution (*P* = 0.657); there were no differences between the right and left sides (48.06 [9.07] versus 47.74 [9.61], respectively); 95% CI: −4.409 to 4.273; *P* = 0.974; paired Student t test.

Additionally, there were no between-side differences in the CP angle in this group of patients.

### LG remodeling assessed by kinesiography (LG angles)

The global mean was 42.95 (11.78), range 19 to 75. The Kolmogorov-Smirnov test did not reject the normal distribution (*P* = 0.601). The LG angles for the right and left sides were 45.20 (11.46) and 40.79 (11.92), respectively; 95% CI: −1.493 to 9.102; *P* = 0.151; paired Student t test. (Table 1).

One LG recording was not included because the lateral jaw motion was guided by the contralateral (nonworking side’s) molar(s). There was no difference in LG angle between the sides in this group of patients.

## Technical validation

### Chewing function assessment

The diagnostic analysis of chewing function requires an objective assessment. Patients should be unaware of the purpose of the observation, and the clinician should be unaware of the patient condition. There are no validated methods to determine whether a subject usually uses one side to chew. Thus, several tests were performed in this study. Observation of the chewing side used in the first cycle was considered sufficient; some authors have reported the observation of seven cycles, whereas other researchers have reported the use of kinesiography recordings. The use of interviews has also been reported. Interviewing a subject about the current chewing side is not adequate because the subject could change the habitual chewing side; thus, both the current and former chewing sides should be addressed in the interview. Use of the same chewing side in all tests allows classification of the subject as a habitual chewer on one specific side; different sides used in any test should be considered alternate chewing.

### CP reliability

Capturing CP recordings requires a methodical clinical procedure. Patients should be instructed to perform lateral jaw movements from the posterior jaw position. Three recordings should be performed on each side; at least two of them that seem the same should be accepted and measured. It is critical to fix the template against the parietal area of the skull, with the horizontal lines parallel to the Frankfort horizontal line^22^. Images were obtained via tele-photography with an objective greater than 100 mm on a digital camera, perpendicular to the image. Two experienced independent assessors measured the angles of the CP tracings with respect to the Frankfort horizontal line. CP measurements are reliable in both intra- and inter-sessional visits^19^. In this study, CP angle measurements showed good interobserver reliability (intra-class correlation coefficient [ICC] = 0.970; 95% CI: 0.945 to 0.983; *P* < 0.001).

### LG reliability

LG angles are reliable measures in healthy subjects^20^ and in patients^21^. In this study, the LG angle measurements showed excellent interobserver reliability (ICC = 0.977; 95% CI: 0.959 to 0.987; *P*<0.001).

## Additional information

**How to cite this article:** López-Cedrún, J. *et al.* Jaw biodynamic data for 24 patients with chronic unilateral temporomandibular disorder. *Sci. Data* 4:170168 doi: 10.1038/sdata.2017.168 (2017).

**Publisher’s note:** Springer Nature remains neutral with regard to jurisdictional claims in published maps and institutional affiliations.

## Supplementary Material



## Figures and Tables

**Table 1:** Subject’s main characteristics and raw data of condylar path and lateral guidance angles.

**Subject**	**Pain side**	**Chewing side**	**Condylar path angles**		**Lateral guidance angles**	
			**right**	**left**	**right**	**left**
1	L	L	43	52	62	45
2	L	A	54	45	42	40
3	L	L	46	53	62	41
4	R	R	41	53	25	28
5	R	R	70	50	50	75
6	R	R	39	35	30	36
7	L	L	60		56	40
8	L	R	40	35	40	30
9	R	R	52	44	45	50
10	L	A	40	39	60	40
11	L	L	44	52	53	43
12	R	1	52	35	34	37
13	L	A	50	60	40	30
14	R	A	39,5	34	19	31
15	L	R	40	46	32	29
16	L	L	45	50	50	45
17	R	R	48	37	52	72
18	L	L	38	51	44	37
19	L	L	55	65	52	35
20	L	A	36	40	55	42
21	R	R		51	45	48
22	L	A	61	70	47	35
23	R	R	62	51		27
24	L	A	50	50	44,5	43
R, right; L, left; A, alternate.						
